# The prevalence of sexual violence during pregnancy in Iran and the world: a ‎systematic review and meta-analysis

**DOI:** 10.5249/jivr.v10i2.954

**Published:** 2018-07

**Authors:** Jafar Bazyar, Hamid Safarpour, Salman Daliri, Arezoo Karimi, Meysam Safi Keykaleh, Mohammad Bazyar

**Affiliations:** ^*a*^Research Center in Emergency and Disaster Health, University of Social Welfare and Rehabilitation Sciences, Tehran, Iran.; ^*b*^Department of Health in Disasters and Emergencies, School of Health, Safety and Environment, Shahid Beheshti University of Medical Sciences, Tehran, Iran.; ^*c*^Department of Epidemiology, Faculty of Public Health, Ilam University of Medical Sciences, ‎Ilam, Iran.; ^*d*^Department of Health Education and Promotion, Faculty of Public Health, Ilam University of ‎ Medical Sciences, Ilam, Iran.

**Keywords:** Iran, Pregnancy, Sexual violence, Meta-analysis, Systematic, Domestic violence

## Abstract

**Background:**

Domestic violence during pregnancy is a public health crisis, because it affects both mother ‎and fetus simultaneously, resulting in irreversible consequences for mothers and their ‎newborns. This study was performed to determine the prevalence of sexual violence during ‎pregnancy in the world and Iran as meta-analysis.‎

**Methods:**

This study is a meta-analysis on the prevalence of sexual violence during pregnancy ‎in the world and Iran that was conducted on Persian and English published articles up to ‎‎2015. To this end, through searching the information by key words and their compounds at SID, Medlib, Irandoc, Google scholar, Pubmid, ‎ISI, Iranmedex, Scopus and Magiran, all related articles ‎were extracted independently by two trained researchers. The results of studies ana-lyzed using ‎the STATA and Spss16 software.‎

**Results:**

In the initial searching of 167 articles, 33 articles related to Iran, 40 articles related to ‎other parts of the world and totally 73 articles met inclusion criteria for study. The prevalence ‎of sexual violence during pregnancy were estimated in the world 17% (CI95%:15% -18%) and ‎in Iran 28% (CI95%: 23% -32%).The prevalence of sexual violence during pregnancy in Iran is ‎‎11 percent more than the world.‎

**Conclusions:**

According to the present meta-analysis results, the prevalence of sexual violence ‎during pregnancy in Iran is high. Given that sexual violence during pregnancy causes damage to ‎the mother and infant, it is recommended that the relevant authorities with the implementation ‎of intervention and educational programs reduce the prevalence of sexual violence during ‎pregnancy.‎

## Introduction

Sexual violence refers to any anti-social behavior that covers touching to sexual assault. This type of violence may occur in the area of private life, marriage and family.^1,2^ Sexual violence occurs in all ages, genders, ethnicities, educational fields and socioeconomic groups and is increasingly on the rise as one of the most important public health problems.^3-5^

According to the World Health Organization (WHO), 35% of women in the world experience physical or sexual violence.^6^ The prevalence of sexual violence in countries with high income is between 3.4 to 11 percent.^7^ In Turkey 33.3% (2003),^8^ Saudi Arabia 6.9% (2011),^9^ Lebanon 26.2% (2007), ^10^ Brazil 3% (2010)^11^ and Mexico 11.3% (2011)^12^ of women were sexually abused during pregnancy. In the study conducted in Iran, the prevalence of sexual violence during pregnancy was estimated to be 25.3% (2015),^13^ which is estimated in Iranian provinces such as Western Azerbaijan 30.2% (2013),^14^ Shiraz 22.3% (2008),^15^ Ahvaz 9.3% (2011)^16^ and Karaj 3.3% (2012) ^17^ of the prevalence of sexual violence was observed during pregnancy.

Women subjected to sexual violence get involved in psychological, nervous and emotional trauma. That affects their whole behavior toward males. Sometimes these women have coldness and depression in their sex lives and never forget hate and fear of man in their family and social life.^18,19^ Pregnancy, for various reasons, such as loss of sexual relations, misconceptions about pregnancy and abnormal sensation of husband about the pregnancy can be a point to start or intensify sexual violence against women.^20^ The woman's feelings during pregnancy, vulnerability of women in this period and increasing economic pressure can be effective factors to increase violence during pregnancy.^21^

Sexual violence can lead to increased incidence of complications related to pregnant women such as acute injuries, Premature rupture of membrane dysfunction, lasting disabilities, eating disorders, sleep disorders, stress disorders, depression, substance abuse and suicide.^22^ Adverse pregnancy outcomes associated with sexual violence is directly caused by sexual or physical trauma or indirectly, such as inserting a stress leads to miscarriage, premature delivery, low-weight birth, premature rupture of membranes, intrauterine growth restriction, perinatal mortality, cesarean delivery and low Apgar score. ^23-25^ For this reason, it is recommended to screen the women for sexual violence at three-month intervals during pregnancy and postpartum.^26^

Sexual violence during pregnancy adversely affects the pregnant women and fetuses that lead to irreparable consequences resulting in huge costs to the health care system.^25^ Knowing the prevalence of sexual violence during pregnancy in the country helps politicians to understand the extent of the problem that can be considered as the first step towards the implementation of interventions for prevention and treatment. The prevalence of sexual violence during pregnancy in different parts of the world and Iran have been reported sporadically and comprehensive information about the general prevalence in the world and Iran is not available to solve this health problem with more appropriate view. This study aimed to determine the prevalence of sexual violence during pregnancy as a systematic review and meta-analysis in the world and Iran in order to estimate the prevalence of this phenomenon in the country and to compare it with the global average, through which the extent of the problem can be realized and appropriate approaches can be adopted to prevent and reduce the incidence of it.

## Methods 

This study was a systematic review and meta-analysis on the prevalence of sexual violence during pregnancy in the world and Iran. The results of this study was obtained based on articles published in Persian and English local and international magazines. In this study, all articles published since 1997 by the end of 2015 were selected during a search in databases of Medlib, SID Scopus, ISI Web of Science, Pubmed, Cochrane, Google scholar, Irandoc, Magiran, Iranmedex. Articles were searched using the Persian keywords of prevalence of violence during pregnancy, sexual violence and domestic violence in Iran and the world individually and combined. In the foreign databases, the words, Violence during pregnancy, Sexual violence, Domestic violence were used.

First, all the papers entitled as sexual violence during pregnancy were collected and after searching completion a list of abstracts was prepared. After hiding the articles information such as author’s name, journal name and etc., full text articles were available to the two expert and trained researchers. Each paper was evaluated independently by them and papers were rejected by both, the reason was mentioned and in case of disagreement between them the article was judged by the third referee. To check the quality of the articles the Strobe check list was used (studies in epidemiology Strengthening the reporting of observational).^27^ This check list has 22 parts that rating was based on the importance of each part according to the study. Final score of the check list was 30 that, the minimum acceptable rating was 15. Required data were extracted using pre-prepared check list containing the sample size, the location, the time of study, type of study and the prevalence of sexual violence.

**Inclusion and exclusion criteria of the studies**

All studies conducted in English and Persian languages in Iran and the world were about sexual violence during pregnancy in all pregnant women and after assessment process papers with the quality rate higher than 20 have met the inclusion criteria. Studies were excluded if they had received score of less than 20 points after the assessment, if were conducted in specific groups (e.g., women with certain diseases, etc.), if did not have sufficient samples, if they had discussed about sexual violence.

**Study Selection**

**Figure 1:**
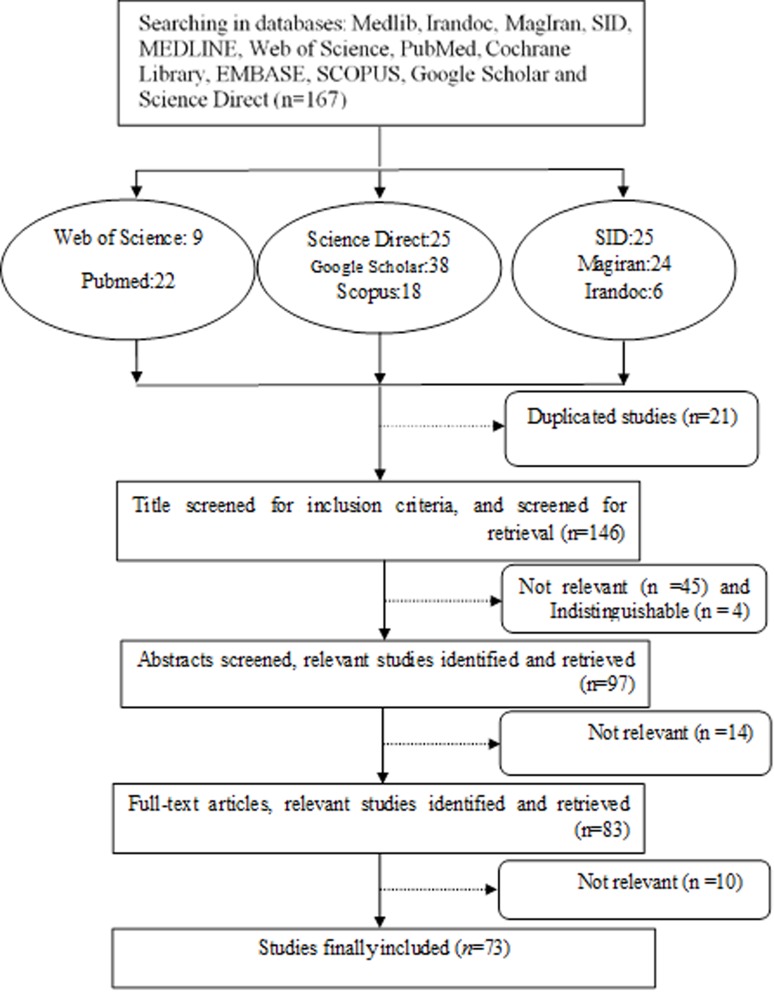
Flowchart of articles entry and selection process for systematic review and meta-analysis.‎

All articles related to the prevalence of sexual violence during pregnancy in the world were included. Accordingly, 167 articles related to sexual violence during pregnancy were found, 21 articles due to replication, 59 articles due to non-relevance were excluded. After reviewing the abstracts, 14 articles lacking the required information and appropriate quality were excluded. Finally, 73 articles met the inclusion criteria and were included into meta-analysis (Figure 1).

**Statistical analysis**

To combine prevalence rates according to the standard deviation of the studies, mean weight was used and considering the exponential distribution of the prevalence, Poisson distribution was considered and Kernel Smoothing method was used for drawing the diagrams.

If there was significant heterogeneity among studies, the random effects model was used; otherwise, the fixed –effects model was used. Also if the confidence interval related to prevalence rate was short, then more weight would be given to specificity.

I^2^ index and Cochran test were used to examine the heterogeneity between the results, Egger's test was used for publication bias, the relationship between the effect size and the year of study with a prevalence rate of meta-regression and for data analysis software STATA (11.1) and SPSS-16 were used.

## Results

**Figure 2:**
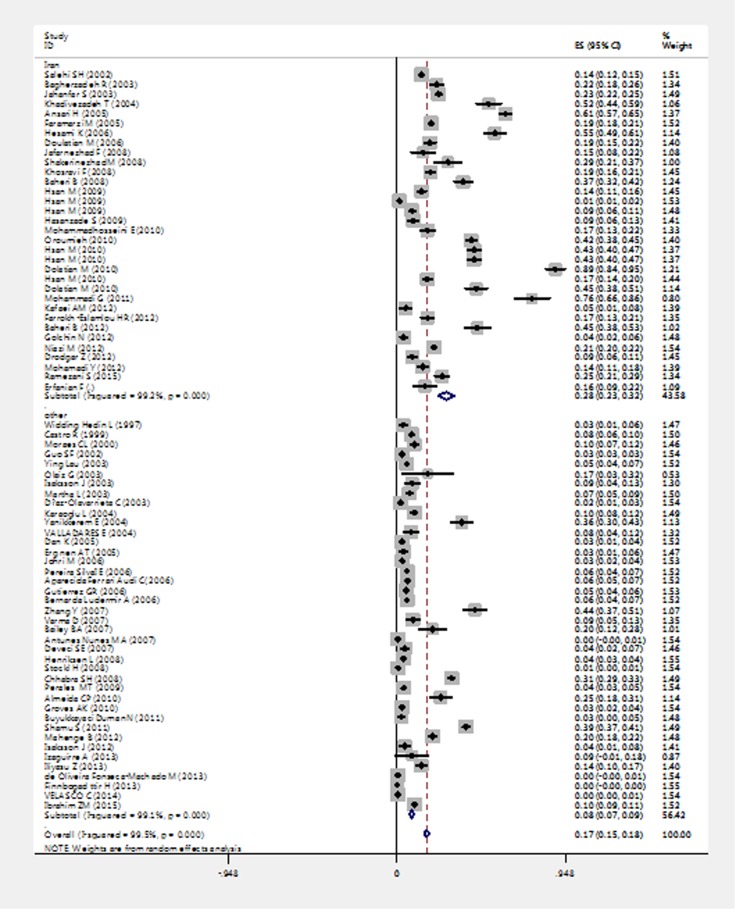
Prevalence of sexual violence during pregnancy and confidence level of 95% in studies in terms of the year and author based on a random effects model. The midpoint of each segment represents the prevalence rate and the segment length measures the confidence level of 95% per study. The diamond mark show that the prevalence rate for all the studies.

In the initial search, of167 articles, 73 articles with sample size of 156209 people met the inclusion criteria,^11-16,28-85^ which contains five case-control studies, 10 cohort studies, 56 cross-sectional studies and 2 meta-analysis studies (Table 1). Average number of samples per study was 2140 people. Articles related to the research subject had been carried out in the period between 1999 and 2015. According to the results of a meta-analysis of studies, the prevalence of sexual violence in Iran was 28% (CI95%: 32% -23%) and in the world it was estimated 17% (CI95%: 18% -15%) (Figure 2).

**Table 1:** General Characteristics of the studied articles that were eligible for the meta-analysis.

Study (Ref)	Year published	Country	Study Population	Age Range (year)	Design	Prevalence of sexual violence %
Ibrahim ZM and et al^28^	2015	Egypt	1875	18-43	Descriptive and analytical	10
Velasco C and et al^29^	2014	Spain	779	20-40	Descriptive and analytical	0.5
de Oliveira and et al^30^	2013	Brazil	358	15-49	Cross-sectional	0.3
Iliyasu Z and et al^31^	2013	Nigeria	400	-	Descriptive and analytical	13.8
Izaguirre A and et al^32^	2013	Spain	35	26-60	Descriptive and analytical	8.5
Finnbogadóttir H and et al^33^	2013	Sweden	1993	18-36	Descriptive and analytical	0.1
Buyukkayaci Duman N and et al^34^	2012	Switzerland	200	18-33	Cross-sectional	2.5
Isaksson J and et al^35^	2012	Nicaragua	147	-	Prospective	4.3
Mahenge B and et al^36^	2012	Tanzania	1180	17-43	Cross-sectional	20
Okour AM^37^	2011	Jordan	303	-	Cross-sectional	15.5
Shamu S and et al^38^	2011	Zimbabwe	2042	15–49	Cross-sectional	38.9
Almeida CP and et al^39^	2010	Portugal	184	-	Cross-sectional	24.6
Groves AK and et al^40^	2010	South Africa	1500	18–46	Cross-sectional	2.8
Akca Toprak Ergonen^41^	2009	Turkey	214	18-45	Prospective	3.4
Perales MT and et al^42^	2009	Peru	2392	-	Cross-sectional	3.9
Stöckl H and et al^43^	2008	Germany	4001	15-35	Cross-sectional	0.7
Henriksen L and et al^44^	2008	Norway	76870	18-45	Prospective	3.5
Antunes Nunes M and et al^11^	2007	Brazil	562	13-42	Prospective	0.5
Varma D and et al^45^	2007	India	203	18-49	Descriptive and analytical	9
Chhabra S and et al ^46^	2007	India	2000	20-49	Descriptive and analytical	30.7
Deveci SE and et al^47^	2007	Turkey	249	-	Descriptive and analytical	4.4
Bailey BA and et al ^48^	2007	America	104	-	Descriptive and analytical	20
Pereira SilvaI E and et al^49^	2006	Brazil	960	18-49	Prospective	6.1
Bernarda A and et al^50^	2006	Brazil	1133	18-49	Prospective	5.7
Aparecida Ferrari Audi C and et al^51^	2006	Brazil	1229	-	Prospective	6.1
Johri M and et al^52^	2006	Guatemala	1263	15-49	Cross-sectional	3
Gutierrez GR and et al ^12^	2006	Mexico	1623	-	Cross-sectional	5
Dan K and et al^53^	2005	Uganda	612	-	Prospective	2.7
Valladares E and et al ^54^	2004	Nicaragua	147	-	Cross-sectional	8
Karaoglu L and et al^55^	2004	Turkey	824	15-49	Cross-sectional	9.7
Yanikkerem E^56^	2006	Turkey	217	-	Cross-sectional	36.4
Martha L and et al^57^	2003	Baltimore	715	18-46	Descriptive and analytical	7.1
Ying Lau and et al^58^	2003	China	1200	-	Cross-sectional	5.5
Olaiz G and et al^59^	2003	Mexico	26	18-49	Cross-sectional	17.3
Díaz-Olavarrieta and et al C^60^	2003	Mexico	1314	13-35	Cross-sectional	1.8
Isaksson J and et al^35^	2003	Nicaragua	147	-	Cross-sectional	8.6
Guo SF and et al^61^	2002	China	12044	19-45	Cross-sectional	2.8
Zhang Y and et al^62^	2007	China	196	-	Cross-sectional	43.8
Moraes CL and et al^63^	2000	Brazil	526	-	Retrospective	9.9
Widding Hedinl and et al^64^	2000	Sweden	207	15-49	Cross-sectional	3.3
Castro R and et al^65^	1999	Mexico	914	18-45	Cross-sectional	8.1
Ramezani S and et al^66^	2015	Shahroud	430	15-43	Cross-sectional	25.3
Drodgar Z and et al^67^	2012	Khorasan	400	15-49	Descriptive and analytical	8.5
B Baheri and et al^17^	2012	Karaj	168	15-49	Descriptive and analytical	45.2
Farrokh-Eslamlou H and et al^68^	2012	Orumieh	350	17-46	Cross-sectional	17.2
Niazi M and et al^13^	2015	Iran	15445	-	Meta-Analysis	21
Golchin N and et al^69^	2012	Golestan	301	15-49	Cross-sectional	3.7
Mohammadi Y and et al^70^	2012	Noorabad	400	-	Descriptive and analytical	14.5
Kafaei AM and et al^71^	2012	Kashan	143	14-42	Descriptive and analytical	4.9
Mohammadi G and et al^72^	2011	Tehran	69	-	Cross-sectional	76
Hasan M and et al^14^	2010	Banab	650	18-39	Cross-sectional	43.4
M Doulatian and et al^25^	2010	Marivan	120	15-49	Prospective	44.6
Hasan M and et al^14^	2013	Miandoab	650	18-39	Cross-sectional	43.4
Hasan M and et al^14^	2013	Mahabad	650	18-39	Cross-sectional	17.1
M Doulatian and et al^25^	2010	Marivan	240	15-49	Prospective	89.2
Mohammadhosseini E and et al^73^	2010	Jahrom	300	15-49	Cross-sectional	17.3
A Ranji and et al^74^	2010	Orumieh	824	15-49	Cross-sectional	41.8
S Hasanzadeh and et al^16^	2009	Ahvaz	300	15-49	Cross-sectional	9.3
Hasan M and et al^14^	2013	Mahabad	650	18-35	Cross-sectional	8.6
Hasan M and et al^75^	2009	Bonab	650	18-35	Cross-sectional	1.5
Hasan M and et al^75^	2009	Miandoab	650	18-35	Cross-sectional	13.8
F Erfanian and et al^76^	2009	Mashhad	109	-	Descriptive and analytical	15.6
Khosravi F and et al^77^	2008	Sanandaj	840	15-49	Cross-sectional	18.8
B Baheri and et al^78^	2008	Karaj	335	15-49	Descriptive and analytical	37.3
Jafarnezhad F and et al^79^	2008	Khorasan	102	15-49	Descriptive and analytical	23.5
Shakerinezhad M and et al^80^	2008	Zanjan	132	15-49	Cross-sectional	28.8
Hesami K and et al^21^	2006	Marivan	243	15-45	Cross-sectional	55.1
Ansari H and et al^81^	2005	Kohgiluyeh	636	-	Retrospective	61
Faramarzi M and et al^82^	2005	Babol	3257	15-49	Descriptive and analytical	19.2
Khadivzadeh T and et al^83^	2004	Mashhad	190	16-38	Descriptive and analytical	51.6
Jahanfar S and et al^84^	2003	Tehran	1800	15-45	Cross-sectional	23.5
Bagherzadeh R and et al^15^	2003	Shiraz	400	15-49	Descriptive and analytical	22.3
Salehi SH and et al^85^	2002	ShahreKord	1600	16-48	Descriptive and analytical	13.8

Among the studies reviewed in Iran, the highest prevalence of sexual violence during pregnancy was related to the study of Dolatian et al. in the city of Marivan in 2011, with the prevalence of 89.2%^25^ and the lowest rate is related to the study conducted by Hassan et al. in city Bonab in 2010 with the prevalence of 1.5%.^76^ In studies conducted in other countries, the highest rate is related to Yong Zhang of China in 2007, with the prevalence of 43.8%^62^ and the lowest is related to Hafrún Finnbogadóttir in Sweden in 2013, with a prevalence rate of 0.1%. ^33^ The world's highest and lowest prevalence was respectively related to the study of Dolatian et al. in 2011 ^25^ and Hafrún Finnbogadóttir in 2007. ^33^

The relationship between the prevalence of sexual violence during pregnancy and the year of the study has been investigated in Figure 3-a. Considering the negative slope of meta-regression diagram (p=0.393) it can be concluded that there is no significant relationship between the years of study and sexual violence. The investigation of the relationship between the sample size and prevalence rate also showed that (Figure 3-b) there is no significant relationship between sample size and prevalence rate (p=0.319). Because it is possible that the studies with more sample size report higher prevalence rate and vice versa. Presented in graphs, circles show the weight of the studies, and the larger the circle, the greater the sample size.

**Figure 3-a:**
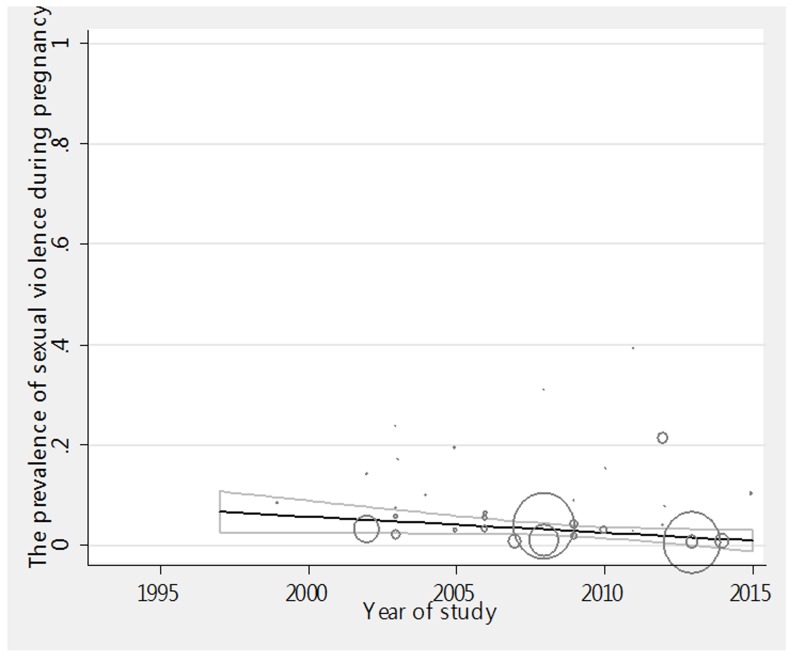
Meta-regression graph of the prevalence of sexual violence during pregnancy in year.

**Figure 3-b:**
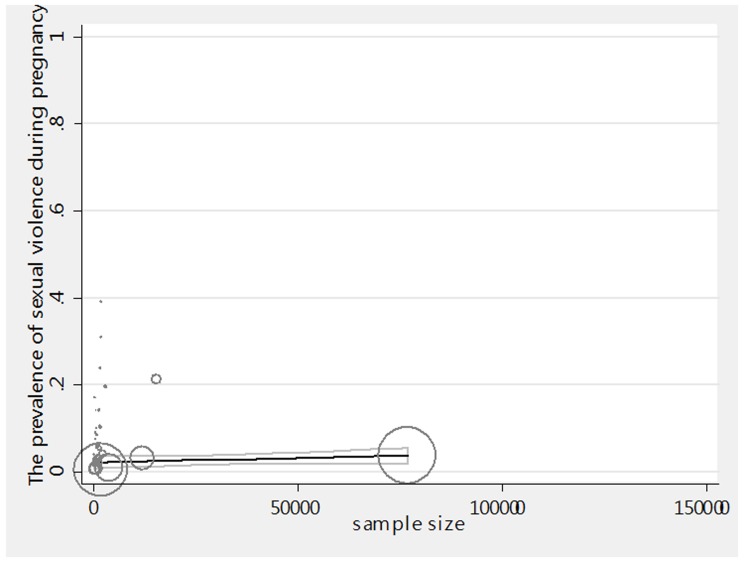
Meta-regression graph of the prevalence of sexual violence during pregnancy in sample size.

## Discussion

The results of a systematic and meta-analysis review of 73 articles related to the subject of research including 32 studies in Iran with the sample number of 41289 during 2002 and 2015 had indicated that the prevalence of sexual violence during pregnancy in Iran is 28 percent. In the study by Niazi et al (2015) conducted as meta-analysis in Iran reported the prevalence of sexual violence in Iran 21% which is less than the value estimated in the present study.^13^ In a study conducted in Turkey (2011) the prevalence of sexual violence during pregnancy was estimated 2.5% which has a low prevalence compared to our country.^34^ The prevalence of sexual violence during pregnancy according to the studies in India (2008)^46^ and China (2013)^62^ was respectively 30.7% and 43.8%, which was higher than the prevalence in Iran and in a study conducted in Egypt (2015)^28^ it was reported as 20% which is close to the prevalence rate in the country of Iran.

Based on meta-analysis of 40 studies conducted in different countries of the world with sample size of 114920 people (by eliminating studies in Iran), the prevalence of sexual violence during pregnancy in the world was 8%. In a study conducted by James L et al. (2013)^86^ as meta-analysis in 2013, the prevalence of sexual violence during pregnancy was 8% which is consistent with our study. According to the findings of the meta-analysis of 73 studies carried out worldwide, the prevalence of sexual violence during pregnancy was 17%. Overall, the prevalence of sexual violence during pregnancy in Iran is 11% higher than the global average.

## Conclusion

The results of this study indicated that sexual violence during pregnancy has high prevalence in the world and Iran and also this rate in Iran has been higher than the global average. Given that sexual violence during pregnancy causes consequences of adverse health effects on the fetus, could adversely affect the physical and mental states of the mother. Therefore, the relevant authorities should reduce the prevalence of the phenomenon with the implementation of intervention programs and training, especially before marriage and pregnancy.

**Acknowledgments**

Hereby, the student research committee of medical university of Ilam who helped us in conducting this study, are sincerely thanked and appreciated.
